# Design and synthesis of new dihydropyrimidine/sulphonamide hybrids as promising anti-inflammatory agents via dual mPGES-1/5-LOX inhibition

**DOI:** 10.3389/fchem.2024.1387923

**Published:** 2024-05-10

**Authors:** Lamya H. Al-Wahaibi, Ali M. Elshamsy, Taha F. S. Ali, Bahaa G. M. Youssif, S. Bräse, Mohamed Abdel-Aziz, Nawal A. El-Koussi

**Affiliations:** ^1^ Department of Chemistry, College of Sciences, Princess Nourah Bint Abdulrahman University, Riyadh, Saudi Arabia; ^2^ Medicinal Chemistry Department, Faculty of Pharmacy, Deraya University, Minya, Egypt; ^3^ Medicinal Chemistry Department, Faculty of Pharmacy, Minia University, Minya, Egypt; ^4^ Department of Pharmaceutical Organic Chemistry, Faculty of Pharmacy, Assiut University, Minya, Egypt; ^5^ Institute of Biological and Chemical Systems, IBCS-FMS, Karlsruhe Institute of Technology, Karlsruhe, Germany; ^6^ Department of Medicinal Chemistry, Faculty of Pharmacy, Assiut University, Assiut, Egypt

**Keywords:** pyrimidine, sulphonamide, inflammation, prostaglandin, lipoxygenase

## Abstract

A novel series of dihydropyrimidine/sulphonamide hybrids **3a–j** with anti-inflammatory properties have been developed and tested as dual mPGES-1/5-LOX inhibitors. *In vitro* assay, results showed that compounds **3c**, **3e**, **3h**, and **3j** were the most effective dual inhibitors of mPGES-1 and 5-LOX activities. Compound **3j** was the most potent dual inhibitor with IC_50_ values of 0.92 µM and 1.98 µM, respectively. *In vivo,* anti-inflammatory studies demonstrated that compounds **3c**, **3e**, **3h,** and **3e** had considerable anti-inflammatory activity, with EI% ranging from 29% to 71%. Compounds **3e** and **3j** were equivalent to celecoxib after the first hour but exhibited stronger anti-inflammatory effects than celecoxib after the third and fifth hours. Moreover, compounds **3e** and **3j** significantly reduced the levels of pro-inflammatory cytokines (PGE_2_, TNF-α, and IL-6) with gastrointestinal safety profiles. Molecular docking simulations explored the most potent derivatives’ binding affinities and interaction patterns within mPGES-1 and 5-LOX active sites. This study disclosed that compound **3j** is a promising anti-inflammatory lead with dual mPGES-1/5-LOX inhibition that deserves further preclinical investigation.

## 1 Introduction

Inflammation is a complex cascade of events that acts as the body’s natural response to injury. It is a crucial aspect of the healing process, helping to fight off infection-causing bacteria, viruses, and other microorganisms. If the acute inflammation fails to fight off the stimulus in time, it may become associated with chronic diseases, such as arthritis, cardiovascular disorders, respiratory diseases, neurodegenerative disorders, and cancer (Megha et al., 2021; Placha and Jampilek, 2021; Harvanová et al., 2023).

Prostaglandin E_2_ (PGE_2_) is a key pro-inflammatory prostanoid involved in many physiological processes, such as pain, inflammation, and fever. That’s why PGE_2_ is overproduced in several inflammatory diseases, such as chronic infections, rheumatoid arthritis, bronchial asthma, and various cancers (Ihsan, 2023). It is produced from arachidonic acid through enzymatic reactions, with microsomal prostaglandin E synthase-1 (mPGES-1) playing a crucial role in its biosynthesis (Zhang et al., 2022). mPGES-1 is highly upregulated in inflammation, making it a potential target for developing selective anti-inflammatory therapies that specifically inhibit PGE_2_ production without affecting other prostaglandins, potentially reducing the risk of gastrointestinal and cardiovascular side effects accompanied by traditional COX inhibitors (Bergqvist et al., 2020). Efforts to progress selective mPGES-1 inhibitors have led to two candidates, LY3023703 (whose trials were halted due to hepatotoxicity) (Jin et al., 2018) and GRC27864 (currently in Phase 2 trials) (Sant et al., 2018).

Another vital enzyme in the inflammatory process is 5-lipoxygenase (5-LOX), which converts arachidonic acid into bioactive leukotrienes. Leukotrienes play roles in various inflammatory conditions, including psoriasis, allergic asthma, and rheumatoid arthritis (Sinha et al., 2019; Meshram et al., 2020). Inhibiting the 5-LOX pathway is seen as a promising tactic for emerging potent anti-inflammatory drugs, although currently, only one 5-LOX inhibitor (Zileuton) is available for treating allergic asthma (Wenzel and Kamada, 1996).

**FIGURE 1:**
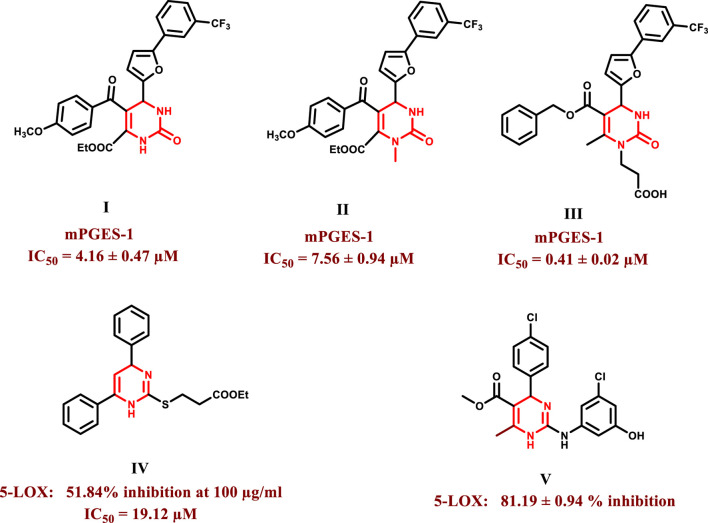
Structures of some dihydropyrimidines as mPGES-1 and 5-LOX inhibitors.

Dihydropyrimidines are an important scaffold in medicinal chemistry because of their diverse variety of biological activities, which include anticancer (Janković et al., 2019; Dowarah et al., 2021), anti-inflammatory (Alfayomy et al., 2021), antioxidant (Vyas et al., 2023), antiviral (Spunde et al., 2022), antibacterial (Zhuang and Ma, 2020), antidiabetic (Jin et al., 2019), and antihypertensive activities (Mahgoub et al., 2021). Over the last few years, compounds possessing dihydropyrimidine moiety have been reported to show potent inhibitory activity against the mPGES-1 enzyme (Figure 1). Compounds I and II were discovered by Lauro et al. as potential mPGES-1 inhibitors by virtual screening with IC_50_ values of 4.16 ± 0.47 μM and 7.56 ± 0.94 μM, respectively (Lauro et al., 2014). Terracciano et al. synthesized compound **III** to optimize further these structures**,** which demonstrated 10-fold higher activity than compound **I** with IC_50_ value in the sub-micromolar range (IC_50_ = 0.41 ± 0.02 μM) (Terracciano et al., 2015). Some dihydropyrimidines were also reported to inhibit the 5-LOX enzyme (Figure 1), such as compound **IV,** designed and synthesized by Lokwani et al. It exhibited 51.84% inhibition of the enzyme at a concentration of 100 μg/mL with an IC_50_ equal to 19.12 μM, which was in line with the computational study in which the carbonyl moiety acted as a metal binding group and established interactions with the ferrous ion in the active site (Lokwani et al., 2015). Another compound, **V**, was developed by Venugopala et al., and it showed promising results demonstrating 81.19% ± 0.94% inhibition at 2.46 µM concentration (Venugopala et al., 2015).

**FIGURE 2:**
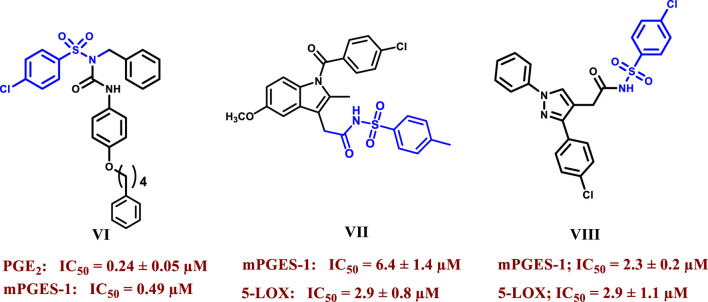
Structures of selected sulphonamides showing mPGES-1 and/or 5-LOX inhibitory activities.

Sulfonamides have drawn much interest due to their widespread application as a privileged scaffold in drug design, with many clinically approved drugs containing this moiety, such as antibacterial (sulfamethoxazole), antidiabetic (gliclazide), anti-inflammatory (celecoxib), diuretic (Bumetanide), antiviral (Dasabuvir), and anticonvulsant drugs (Sultiame) (Apaydın and Török, 2019; Yousif et al., 2022). An example of an anti-inflammatory sulphonamide acting as anti-mPGES-1 is compound VI, which Kim et al. synthesized with the ability to inhibit PGE_2_ production in A549 cells at an IC_50_ of 0.24 μM which was about 9-fold more active than the standard inhibitor MK-886 (Figure 2) (Kim et al., 2021). Elkady et al. reported that replacement of the carboxylic group of NSAIDs with a substituted benzene sulphonamide group yielded compounds with dual mPGES-1/5-LOX inhibition and decreased COX inhibition compared to the parent drugs, such as indomethacin derivative **VII,** which showed IC_50_ values of 6.4 µM and 2.9 µM for mPGES-1 and 5-LOX respectively (more than six fold more potent mPGES-1 inhibitor than indomethacin) and lonazolac derivative **VIII** which showed IC_50_ values of 2.3 µM and 2.9 µM for mPGES-1 and 5-LOX respectively (19 and 20 folds more potent than lonazolac calcium against mPGES-1 and 5-LOX respectively) (Figure 2) (Elkady et al., 2012).

### 1.1 Rationale for design

**FIGURE 3:**
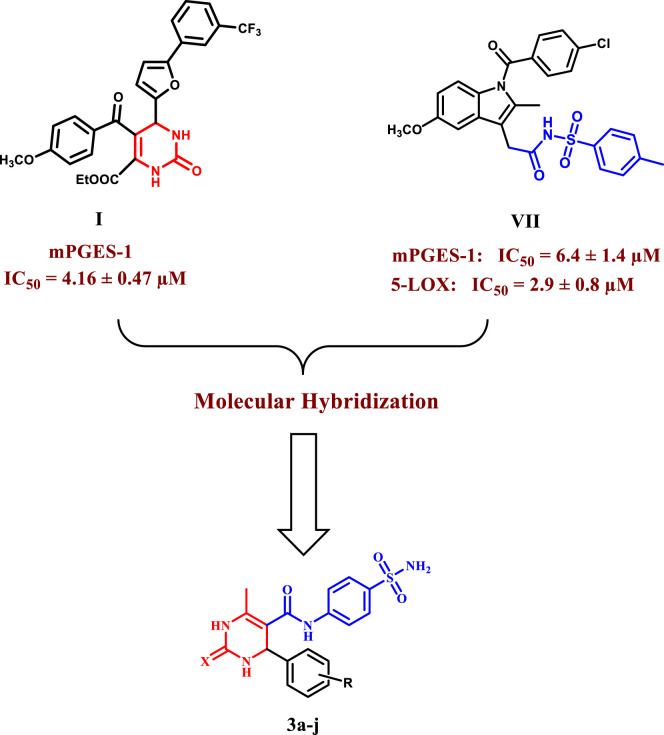
Design of the target compounds **3a–j** as dual mPGES-1/5-LOX inhibitors.

As part of our ongoing search for a highly safe and effective anti-inflammatory drug (Elbastawesy et al., 2015; Abdelazeem et al., 2017; Abdelrahman et al., 2017; Youssif et al., 2019; Abdel-Aziz et al., 2021; Hendawy et al., 2021; Mohassab et al., 2021; Abdel et al., 2022; Shawky et al., 2023), we aimed to fill the research gap on the limited investigation of dihydropyrimidines’ potential as dual mPGES-1 and 5-LOX inhibitors in the current study. Our main objective was to explore the anti-inflammatory properties of novel dihydropyrimidine/sulfonamide hybrids **(3a–j)**, taking advantage of the known anti-inflammatory potencies of both components. By combining these two important scaffolds into a single molecule, we aimed to investigate the potential synergistic effects and enhanced anti-inflammatory activity. Although previous research has examined the inhibitory potential of each scaffold individually, investigating these hybrid compounds is relatively new and holds promising prospects for developing more effective anti-inflammatory agents. Moreover, the synthesized dihydropyrimidine/sulfonamide derivatives were designed with various substitutions of electron-donating and electron-withdrawing groups to investigate their structure-activity relationship (SAR). The most effective derivatives were further subjected to molecular docking and dynamic simulations to explore their interactions within the active sites of mPGES-1 and 5-LOX (Figure 3).

## 2 Results and discussion

### 2.1 Chemistry

**SCHEME 1:**
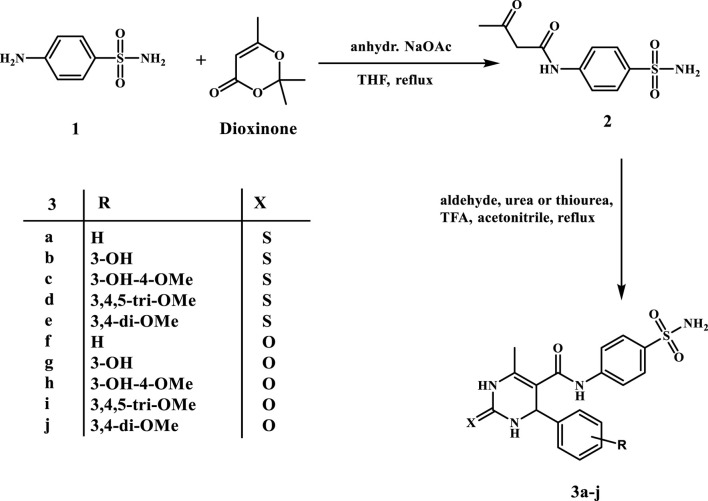
Synthesis of pyrimidine-5-carboxamides **3a–j**.

Scheme 1 shows the chemical synthesis of target compounds **3a–j**. The first step entails a 16-h reaction of sulfanilamide with a slight excess of 2, 2, 6-trimethyl-4*H*-1, 3-dioxin-4-one (Dioxinone) in a small amount of refluxing THF in the presence of anhydrous sodium acetate. Compound **2**’s structure was confirmed by its reported melting point (Fares et al., 2020). Pyrimidine-5-carboxamides **3a–j** were synthesized via acid-catalysed Biginelli cyclo-condensation of the intermediate **2** with various substituted benzaldehydes in the presence of urea or thiourea. The superlative yields were obtained by heating the reaction mixture in acetonitrile under reflux with a catalytic amount of trifluoroacetic acid for 18 h.

^1^H NMR, ^13^C NMR, mass spectra and elemental microanalysis confirmed the chemical structures of the target compounds **3a–j**. All compounds showed a doublet at δ 5.31–5.44 ppm (CH) and a singlet at δ 2.05–2.09 ppm (CH_3_), confirming the formation of the dihydropyrimidine derivative. In compounds **3b–e** and **3g–j**, the sulfamoyl NH_2_ group showed as a singlet at δ 7.21–7.23 ppm or as a multiplet with the aromatic protons of the unsubstituted phenyl ring in compounds **3a** and **3f**. ^13^C NMR DEPTQ-135 spectra of the title compounds showed characteristic (CH_3_) peak at δ 16.54–16.62 ppm and (CH) peak at δ 54.60–55.00 ppm, while the aromatic carbons of the two phenyl rings appeared at δ 110.37–153.00 ppm. Furthermore, compounds **3a–e** containing dihydropyrimidine-thione scaffold showed a highly downfield shifted peak at 173.85–174.38 ppm, which corresponds to the C2 thione moiety, while compounds **3f-j** containing a dihydropyrimidinone nucleus showed characteristic C2 carbonyl peak at δ 152 ppm. Their ESI+ and ESI- mass spectra further confirmed the compounds, which showed characteristic [M + Na]^+^ and [M-H]^−^ peaks for the synthesized compounds.

### 2.2 Biology

#### 2.2.1 Microsomal PGES-1 (mPGES-1) enzyme assay

A cell-free assay was conducted to evaluate the capacity of compounds **3a–g** to act as inhibitors of mPGES-1. In this assay, microsomal fractions from IL-1β-stimulated A549 cells served as the enzyme source (Gürses et al., 2021). During the initial screening phase, compounds **3a–j** were examined for their effects on mPGES-1 at a concentration of 10 µM. The residual activity percentage (RA %) was determined for each target compound, as depicted in Table 1. Remarkably, compounds **3c**, **3e**, **3h**, and **3j** extremely inhibited mPGES-1 activity with RA% ranging from 24.7 to 33.6, but none of the other compounds were significantly active at 10 µM. A more thorough analysis of the IC_50_ values for **3c**, **3e**, **3h**, and **3j** revealed values between 0.92 and 1.5 µM (Table 1), significantly outperforming the reference MK886 (IC_50_ = 2.2 µM). Compound **3j** (*R* = 3, 4-di-OMe, *X* = O) was the most active analog, with an IC_50_ value of 0.92 µM being 2.4-fold more potent than the reference MK886. Compound **3e** (*R* = 3,4-di-OMe, *X* = S), which substitutes sulfur for oxygen at position C2 of **3j**, had an IC_50_ of 0.97 µM, demonstrating that both oxygen and sulfur atoms at position C2 were tolerated for inhibitory activity against mPGES-1. Compounds **3h** (*R* = 3-OH-4-OMe, *X* = O) and **3c** (*R* = 3-OH-4-OMe, *X* = *S*) demonstrated comparable IC_50_ values of 1.32 µM and 1.53 µM, respectively. These compounds were 1.5-fold less potent than **3j**, implying that the 3,4-di-OMe group may significantly influence the mPGES-1 inhibitory activity in this chemotype. Another intriguing finding was that changing the 3, 4-diOMe group in **3j** to the 3,4,5-trimethoxy group caused the analog **3i** (*R* = 3,4,5-tri-OMe, *X* = O) to be three times less potent than **3j**, indicating the importance of the methoxy group numbers for activity.

**TABLE 1:** Inhibition of mPGES-1/5-LOX assay of compounds 3a–j.

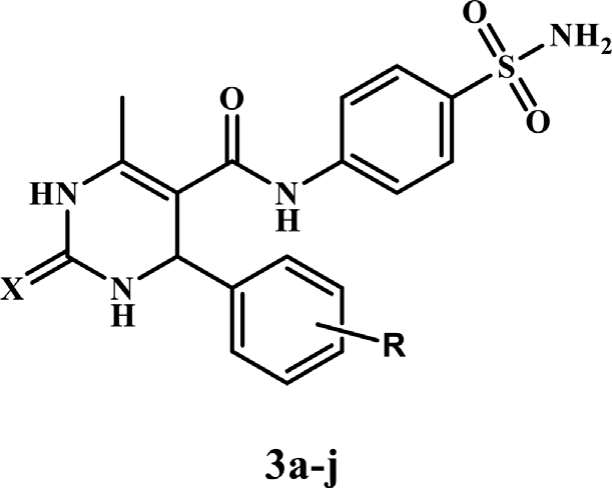					
Compound	R	X	mPGES-1 RA (%) 10 µM	mPGES-1 IC_50_ µM	5-LOX IC_50_ µM
3a	H	S	89.2	4.28	5.85
3b	3-OH	S	69.2	3.89	4.82
3c	3-OH-4-OMe	S	33.6	1.53	2.87
3 d	3, 4, 5-trimethoxy	S	46.8	2.70	3.65
3e	3, 4-di-OMe	S	28.6	0.97	2.07
3f	H	O	91.4	4.78	5.34
3 g	3-OH	O	64.7	3.45	4.45
3 h	3-OH-4-OMe	O	31.2	1.32	2.64
3i	3, 4, 5-trimethoxy	O	53.6	2.89	3.97
3j	3, 4-di-OMe	O	24.7	0.92	1.89
MK886				2.2	--
Meclofenamate	—	—	—	--	5.64

The unsubstituted derivatives **3a** (*R* = H, *X* = S) and **3f** (*R* = H, *X* = O), with IC_50_ values of 4.28 µM and 4.78 µM, respectively, were the least potent, indicating that the substitution at C4 Phenyl group is essential for activity and that the activity was increased in the following order: 3, 4-diOMe > 3-OH-4-OMe >3, 4, 5-trimethoxy > 3-OH > H.

#### 2.2.2 5-LOX enzyme assay

The capacity of compounds 3a–j to inhibit the enzyme 5-lipoxygenase (5-LOX) has been investigated (Youssif et al., 2019). The IC_50_ of each compound is listed in Table 1.

The results of this assay matched the results of the m-PGES-1 inhibitory assay, in which compound 3j (*R* = 3,4-di-OMe, *X* = O), the most potent m-PGES-1 inhibitor, was found to be the most active as a 5-LOX inhibitor, with an IC_50_ value of 1.89 µM compared to the reference IC_50_ value of 5.60 µM. Once again, compound **3e** (*R* = 3, 4-di-OMe, *X* = S) was ranked second in activity as a 5-LOX inhibitor with an IC_50_ value of 2.07 µM. According to the data on biological activity, **3j** is the most effective dual inhibitor of mPGES-1 and 5-LOX activities. Compounds **3c**, **3e**, and **3h** are potent inhibitors of mPGES-1 and 5-LOX, while the remaining compounds have moderate to weak inhibitory activity against both targets.

#### 2.2.3 Assay for anti-inflammatory action

Compounds **3c**, **3e**, **3h**, and **3j**, the most effective dual m-PGES-1/5-LOX inhibitors, were chosen to be investigated for *in vivo* anti-inflammatory activity using the carrageen-induced paw edema bioassay method devised by Winter et al. (Winter et al., 1962). The compounds’ efficacy was measured as edema inhibition percentage (EI %) after 1, 3, and 5 h of carrageenan injection vs. the conventional medicine Celecoxib. Results are cited in Table 2. The findings revealed that the studied compounds have significant anti-inflammatory properties, with EI% ranging from 29% to 71%.

**TABLE 2:** Anti-inflammatory impact of **3c**, **3e**, **3h**, and **3j**.

Compound no.	Baseline	% Of edema inhibition		
	Paw diameter (mm) ±SE	1 h	3 h	5 h
Control	2.80 ± 0.09	—	—	—
Celecoxib	2.10 ± 0.07	40	54	22
**3c**	2.30 ± 0.06	29	46	55
**3e**	2.10 ± 0.09	35	57	65
**3h**	2.25 ± 0.06	30	49	58
**3j**	2.05 ± 0.09	38	60	71

After 5 h of treatment, all evaluated compounds showed greater anti-inflammatory effects than Celecoxib. They showed a rapid onset of action and a long-lasting effect until the fifth hour after the compounds were delivered. Compounds **3e** and **3j** were comparable to celecoxib after the first hour but had greater anti-inflammatory effects than celecoxib after the third and fifth hours (Table 2). According to our findings, the novel scaffold is a plausible lead for building highly effective m-PGES-1/5-LOX dual inhibitors as prospective anti-inflammatory medicines.

#### 2.2.4 Effect on inflammatory cytokines

##### 2.2.4.1 Prostaglandin E_2_ (PGE_2_)

Inhibiting PGE_2_ is a crucial strategy in anti-inflammatory therapy, playing a pivotal role in managing inflammation and its associated conditions. PGE_2_, a potent inflammatory mediator, is highly detected in inflammatory diseases (Fattahi and Mirshafiey, 2012; Hassan et al., 2019). Moreover, recent research has demonstrated the importance of PGE_2_ reduction in anti-inflammatory actions (Cardoso et al., 2020). To assess the potential of compounds **3e**, **3h**, and **3j** to inhibit PGE_2_, the levels of PGE_2_ in serum samples taken 4 hours after administering subcutaneous carrageenan injections were measured. The percentage of PGE_2_ inhibition was determined, and the values are presented in Table 3.

**TABLE 3:** Rat serum concentrations of PGE_2_, TNF-α and IL-6 for compounds **3e**, **3h, 3j** and Meloxicam.

Compound	Inflammatory markers [serum concentration in pg/mL, %inhibition]					
	PGE_2_		TNFα		IL-6	
3e	83.50 ± 2.30^b^	73	78.10 ± 2.20^b^	67	94.10 ± 2.75^b^	75
3 h	95.00 ± 2.50^abc^	69	102.70 ± 2.90^ab^	56	140.70 ± 4.20^ab^	63
3j	62.60 ± 2.75^b^	79	68.50 ± 2.00^bc^	71	85.50 ± 2.35^bc^	77
Meloxicam	82.50 ± 2.58^b^	73	88.50 ± 2.40^ab^	62	114.01 ± 2.82^ab^	70
Control (pre)	71.10 ± 1.05	ND	44.60 ± 1.30	ND	74.1 ± 2.71	ND
Control (post)	301.50 ± 11.70^a^	ND	234.60 ± 4.20^a^	ND	376.10 ± 13.7^a^	ND

Data are expressed as (mean ± SE). Statistics were done by One-way ANOVA, and confirmed by Tukey’s test. Carr; carrageenan, Melox; meloxicam, PGE_2_; Prostaglandin E_2_, IL-6; Interleukin 6, TNF-α; Tumor necrosis factor α.

^a^ *p* < 0.05: Statistically significant from control (pre) group.

^b^ *p* < 0.05: Statistically significant from control (post) group (Carrageenan).

^c^ *p* < 0.05: Statistically significant from standard group (Meloxicam).

The results of this testing were in line with the *in vitro* findings. Compared to the reference drug meloxicam, which displayed a 72.60% inhibition of PGE_2_, all three compounds examined exhibited marked reductions in serum PGE_2_ levels, ranging from 68.50% to 79.20%. Notably, compounds **3e** and **3j** demonstrated the highest activity, inhibiting PGE_2_ by 72.70% and 79.20%, respectively. It is worth mentioning that these same compounds were also the most potent dual mPGES-1/5-LOX inhibitors.

##### 2.2.4.2 Determination of rat serum TNF-α and IL-6

TNF-α and IL-6, the pro-inflammatory cytokines, are pivotal in promoting inflammation and are often associated with developing chronic illnesses (Hunter and Jones, 2015). Decreased plasma levels of these mediators play a significant role in achieving an overall anti-inflammatory effect, which, in turn, helps mitigate the progression and severity of various chronic conditions (Desai and Furst, 2006). In the current study, we assessed the serum concentrations of TNF-α and IL-6 in the blood samples collected from rats following administration of compounds **3e**, **3h**, and **3j**, as presented in Table 3. All tested compounds significantly reduced the concentrations of TNF-α (% inhibition = 56–71) and IL-6 (% inhibition = 63–77) in rat serum. Notably, compound **3j** demonstrated the highest efficacy, with a TNF-α % inhibition of 71%, surpassing that of the reference drug meloxicam (%TNF-α inhibition = 62) and exhibiting a higher drop in serum IL-6 levels (% inhibition = 77), in comparison to meloxicam (% IL-6 inhibition = 70).

#### 2.2.5 Gastric ulcerogenic activity

The two most common side effects of long-term NSAID use are gastrointestinal erosion and ulcers (Hendawy et al., 2021). As a result, we were curious about the ulcerogenic potential of the most efficacious drugs, **3e** and **3j**, when given orally. The ulcerogenic effects of **3e** and **3j** were assessed by macroscopic inspection of rat intestinal mucosa after oral administration of 10 mg/kg of **3e**, **3j**, indomethacin, and celecoxib (Manivannan and Chaturvedi, 2011).

Compound **3j** did not generate ulceration in the isolated rat stomach, whereas compound **3e** produced mild hyperemia but no widespread ulceration (Table 4). Compounds **3e** and **3j** were found to have a potent m-PGES-1/5-LOX inhibitory profile with no (or weak) gastrointestinal side effects.

**TABLE 4:** Ulcerogenic effects of compounds **3e** and **3j**.

Groups	Score	
	No. of gastric ulcers	Severity lesions
Control	0	0
3e	0.60 ± 0.01	1.00 ± 0.01
3j	0	0
Celecoxib	2.5 ± 0.10	5.80 ± 0.20
Indomethacin	8.5 ± 0.40	12.50 ± 0.70

### 2.3 Molecular docking studies

**FIGURE 4:**
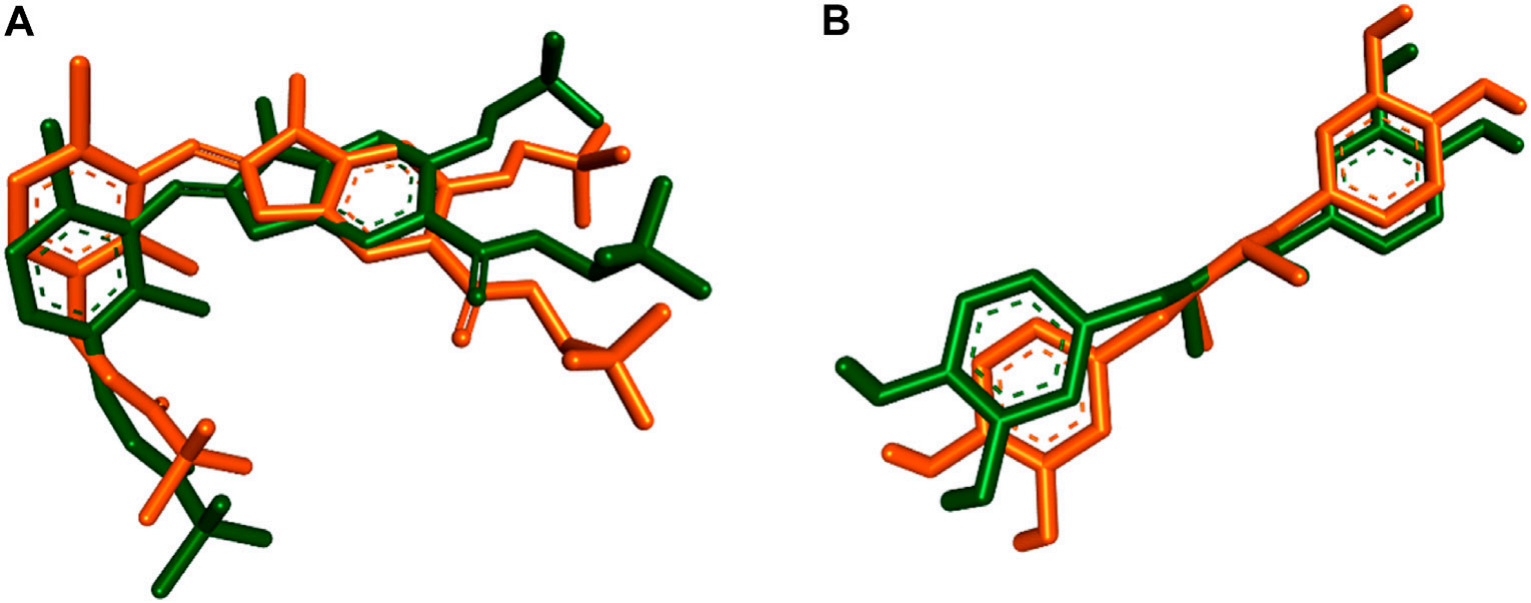
**(A)** and **(B)** Superposition of both the redocked poses and co-crystallized inhibitors inside mPGES-1 (PDB ID, 4 bpm) and 5-LOX (PDB ID, 6 n2w) respectively.

**FIGURE 5:**
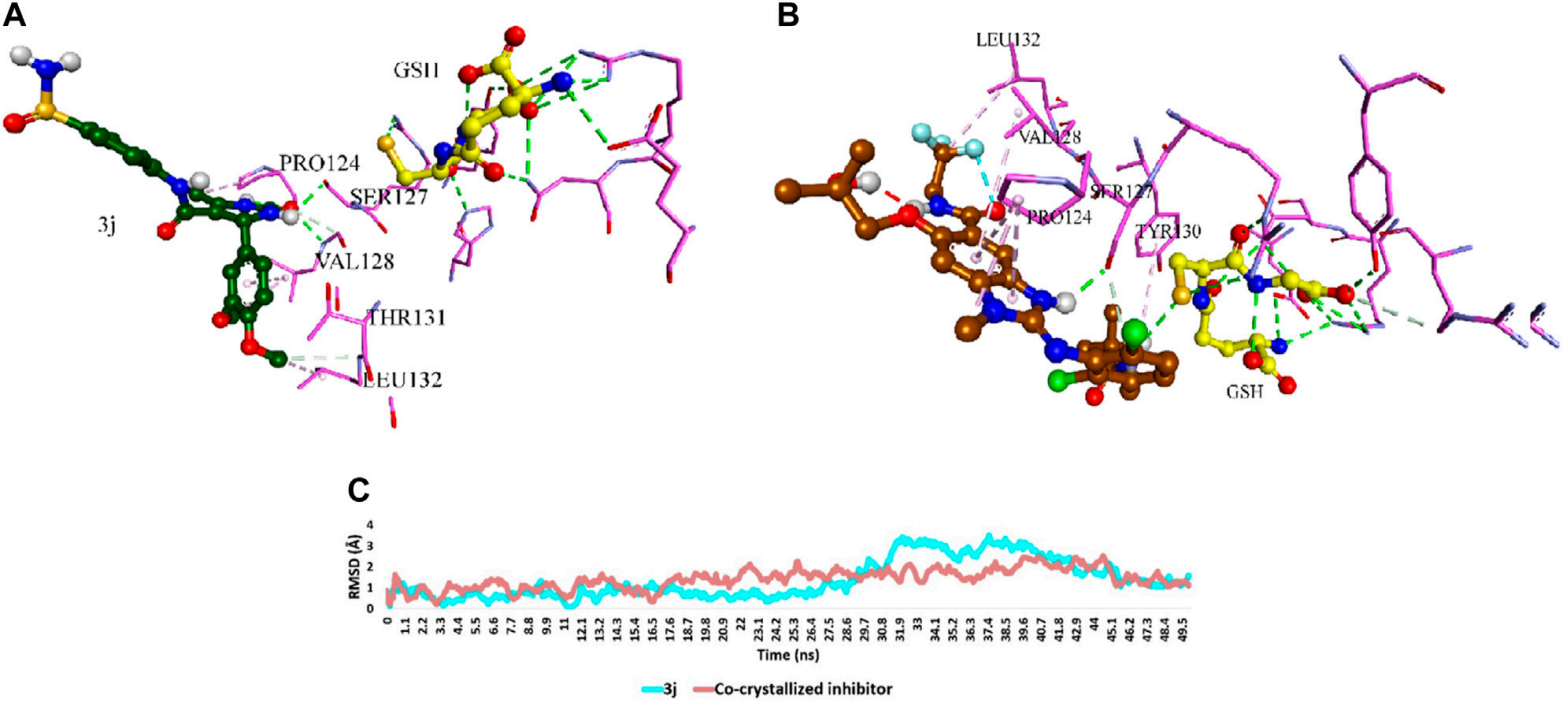
**(A)** and **(B)** Binding mode of **3j** inside the co-crystallized inhibitor-binding site of mPGES-1 (PDB ID: 4bpm) in comparison with that of the co-crystallized inhibitor, respectively. **(C)** RMSDs of **3j** inside the co-crystallized inhibitor-binding site of mPGES-1 in comparison with that of the co-crystallized inhibitor over 50 ns-long MD simulations.

To explore the potential interactions of compound **3j** with the target proteins mPGES-1 and 5-LO, we created their structural models and performed molecular docking simulations using the crystalline structures of these proteins (PDB ID, 4 bpm and 6 n2w, respectively) as reported by Li et al, (2014) and Gilbert et al, (2020). For mPGES-1 (PDB ID, 4 bpm), our strategy involved docking compound **3j** at the site occupied by a co-crystallized inhibitor, rather than the glutathione (GSH) binding site, due to the latter’s strong affinity and resistance to displacement by other inhibitors, as discussed by Li et al, (2014) and Koeberle and Werz, (2018). The docking protocol was validated by re-docking the co-crystalized ligands into the active sites of both enzymes (i.e., mPGES-1 and 5-LO, respectively). The resulting top-scoring poses of both ligands were in good alignment with the co-crystalized ones with slight deviations (RMSDs = 1.27 and 1.04, respectively). Superposition of the co-crystallized ligands of both enzymes is illustrated in Figure 4. The docking results, illustrated in Figure 5, reveal that the preferred orientation of **3j** was comparable to the binding behavior of the co-crystallized inhibitor, engaging in a hydrogen bond with SER-127 and hydrophobic contacts with LEU-132 and PRO-124, alongside an additional hydrogen bond with PRO-124’s backbone.

**FIGURE 6:**
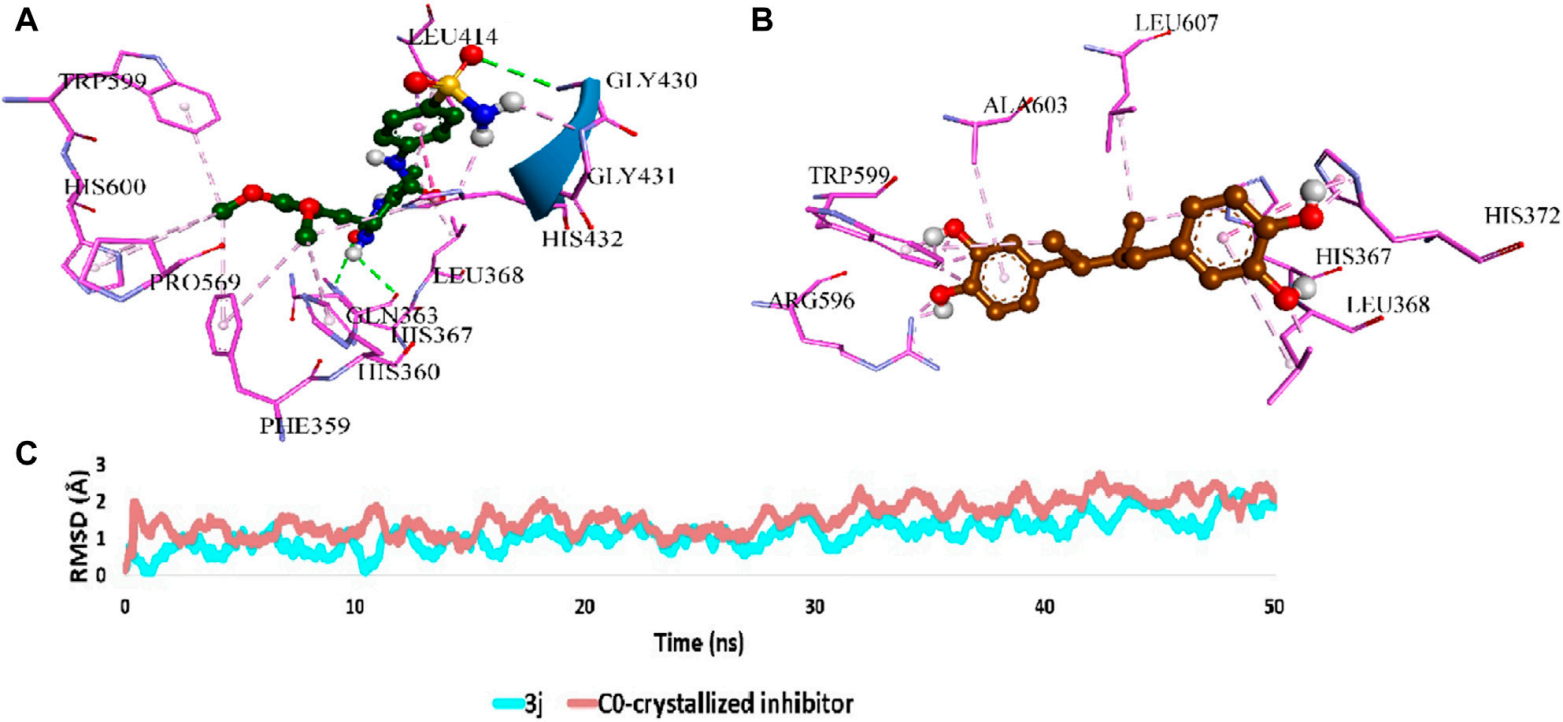
**(A)** and **(B)** Binding mode of **3j** inside the redox binding site of 5-LO (PDB ID, 6n2w) in comparison with that of the co-crystallized inhibitor, respectively. **(C)** RMSDs of **3j** inside the redox binding site of 5-LO in comparison with that of the co-crystallized inhibitor over 50 ns-long MD simulations.

Regarding 5-LO (PDB ID: 6n2w), **3j** was docked into the enzyme’s redox site, achieving a binding posture partially akin to that of the native inhibitor, as depicted in Figure 6. Here, **3j** predominantly formed hydrophobic interactions with residues LEU-368, PHE-359, LEU-414, and TRP-599, while also establishing hydrogen bonds with GLY-430 and HIS-432.

### 2.4 Molecular dynamics simulations

To validate the docking poses of the most potent compound **3j** inside the active sites of both mPGES-1 (PDB ID: 4bpm) and 5-LO (PDB ID: 6n2w), respectively, they were subjected to 50 ns-long molecular dynamic simulations (MDS). As shown in Figures 5C, 6C, **3j** exhibited acceptable stability inside each binding site throughout the simulation with an average RMSD of 2.2 Å and 1.4 Å, respectively relative to the initial docking poses.

**FIGURE 7:**
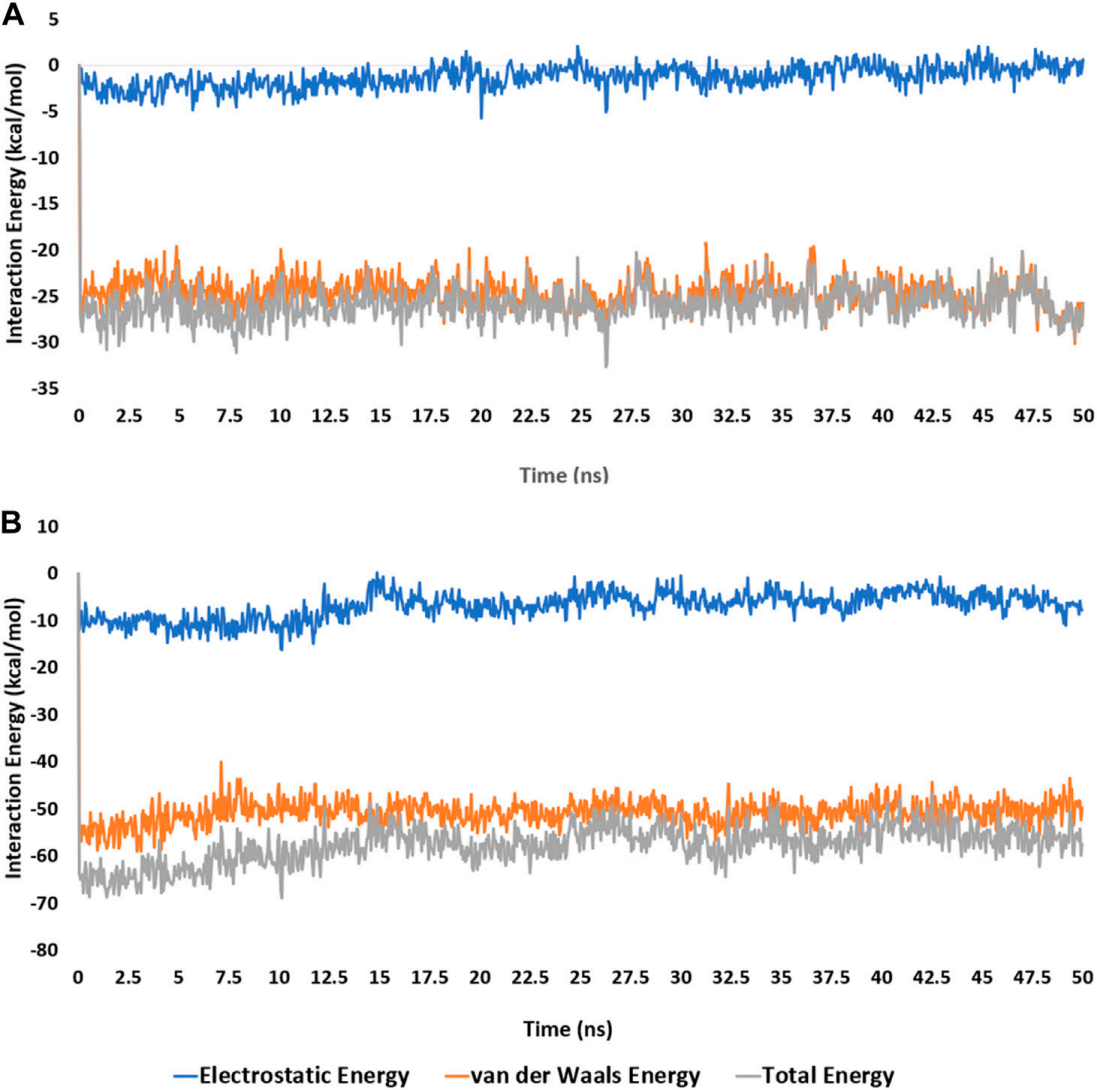
Electrostatic and van der Waals Interaction energies of compound **3j** inside the active sites of mPGES-1 and 5-LO over 50 ns-long MD simulations [**(A)** and **(B)**, respectively].

Accordingly, the calculated electrostatic and van der Waals interaction energies of **3j** within the active site of each enzyme showed an average total interaction energies of around −61.19 and −26.52 kcal/mol, respectively (Figure 7). Moreover, their co binding free energies (Δ*G*_Binding_) using MM-PBSA were found to be −16.8422 and −7.998 kcal/mol, respectively indicating strong affinities towards the corresponding active sites, particularly with 5-LOX (Table 5).

**TABLE 5:** Calculated binding free energies (Δ*G*_Binding_; MM-PBSA) of compound **3j** in complex with 5-LO and mPGES-1. The values were calculated in kcal/mol.

Energy component	3j**-5-LO**	3j**-mPGES-1**
Δ*G*_gas_	−24.9867	−20.7645
Δ*G*_solv_	8.1445	12.7665
Δ*G*_Total_	−16.8422	−7.998

**FIGURE 8:**
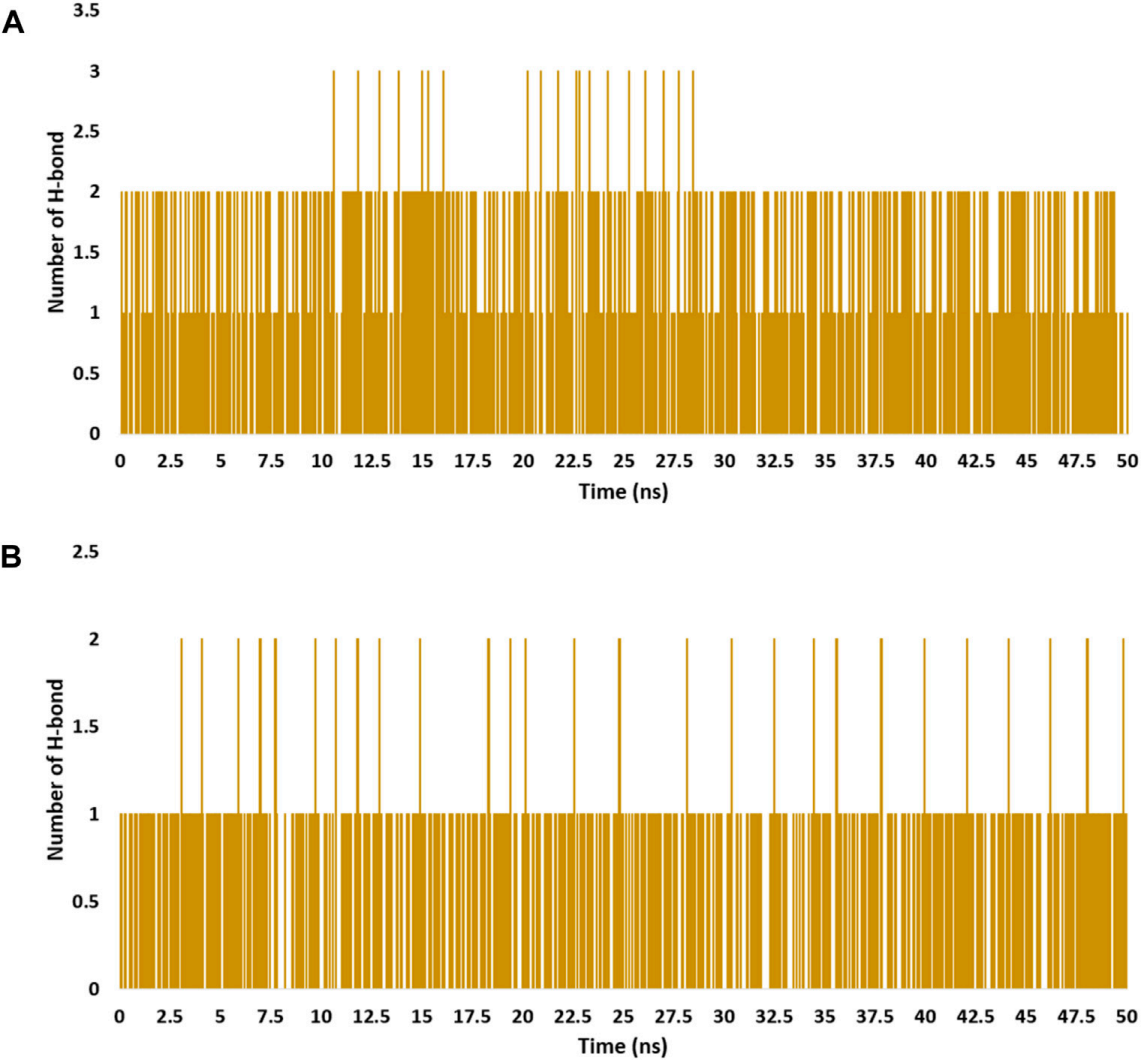
Number of H-bonds detected for **3j** inside the active sites of mPGES-1 and 5-LO over 50 ns-long MD simulations [**(A)** and **(B)**, respectively]. Cut-off distance for H-bonds was set to 3.0 Å.

Compound **3j** established stable multiple hydrophilic and hydrophobic interactions, particularly H-bonds that were found to be around 2 H-bonds inside 5-LOX, and around one H-bond inside mPGES-1 throughout the simulation course (Figure 8).

In conclusion, compound **3j** exhibited acceptable levels of binding stability inside the active sites of both 5-LO and mPGES-1 throughout a 50-ns long MDS indicating a possible inhibitory activity against both enzymes.

### 2.5 Structure-activity relationship (SAR) of compounds 3a–j

SAR studies could be summarized as follows.

• Substitution on the C4 phenyl ring on the DHPM scaffold proved advantageous for both mPGES-1 and 5-LOX inhibitory activities, with the unsubstituted compounds **3a** and **3f** being the least active of the series.
• The number of methoxy groups greatly affected the activity, with the highest potency exhibited by the 3, 4 dimethoxy derivatives **3e** and **3j.**
• On the other hand, the trimethoxy derivatives **3d** and **3i** were less potent against both mPGES-1 and 5-LOX (possibly due to the increased steric hindrance).
• The introduction of sulfonamide group was beneficial as it provided auxiliary interactions with GLY-430 and HIS-432 in the 5-LOX redox binding site through hydrogen bonding. which further stabilized its binding.
• The enzymes tolerated both urea and thiourea moieties well, with urea derivatives having slightly better activity against both enzymes.
• The DHPM anchored compound **3j** to the mPGES-1 active site through formation of important hydrogen bonding with SER-127 and PRO-124

**FIGURE 9:**
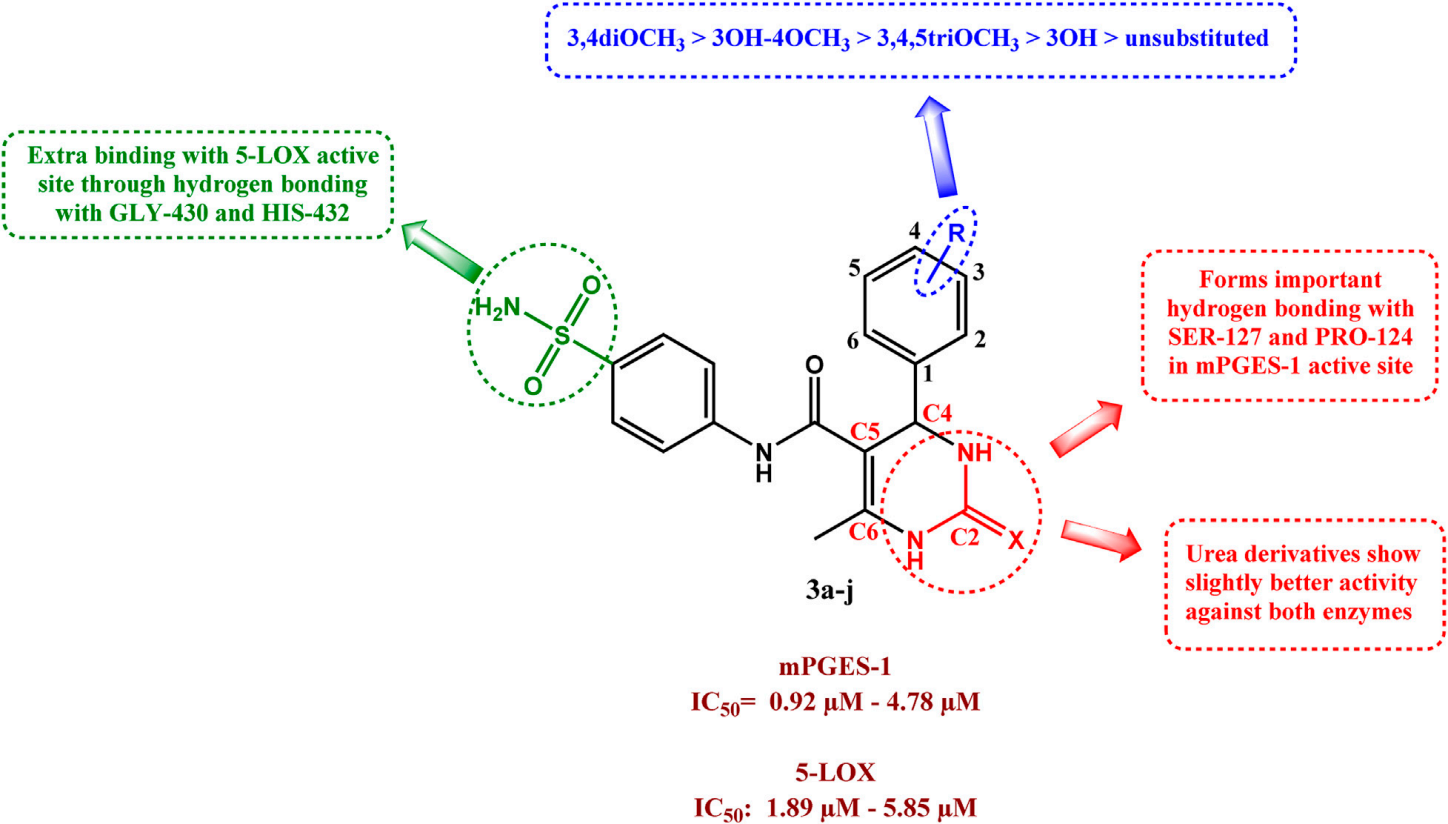
SAR of compounds **3a–j** as dual mPGES-1/5-LOX inhibitors.

SAR of compounds 3a**–**j as dual mPGES-1/5-LOX inhibitors is outlined in Figure 9.

## 3 Conclusion

As potential anti-inflammatory agents, a novel class of dual mPGES-1/5-LOX inhibitors **3a–j** has been developed and tested *in vitro*. Compounds **3c**, **3e**, and **3j** were discovered to be effective mPGES-1 and 5-LOX inhibitors. The most potent dual inhibitor of mPGES-1 and 5-LOX activity was **3j**. Compounds **3c**, **3e**, and **3j** showed promising anti-inflammatory action with rapid onset of action and long-lasting effects up to 5 h with no or weak gastrointestinal unwanted side effects. The levels of pro-inflammatory cytokines (PGE_2_, TNF-α, IL-6) also decreased significantly. Furthermore, molecular docking studies predicted the binding affinities and interaction patterns of these compounds with both mPGES-1 and 5-LOX, which revealed that these compounds established key interactions with both targets with better affinities than the cocrystallized ligands. The most potent derivatives will be subjected to more detailed biological assays to evaluate their anti-inflammatory activity to obtain a lead compound for future optimization.

## 4 Materials and methods

### 4.1 Chemistry

**General details:** (See supplementary data)

Sulfanilamide, 2, 2, 6-trimethyl-4*H*-1, 3-dioxin-4-one (Dioxinone), and all solvents were purchased from Sigma Aldrich, Combi-Blocks, Fisher Scientific and they were used without purification unless mentioned.

#### 4.1.1 General procedure for synthesis of 4-aryl-6-methyl-N-[4-sulfamoylphenyl]-2-oxo/thioxo-1,2,3,4-tetrahydropyrimidine-5-carboxamides (3a**–**j)

A mixture of the appropriate aldehyde (2 mmol), urea or thiourea (3 mmol, 0.228 g), and compound **2** (2 mmol, 0.512 g) were heated in acetonitrile containing a catalytic amount of trifluoroacetic acid (0.4 mmol, 30 µL) for 8 h. The excess solvent was evaporated, and the reaction mixture was left overnight. The solid precipitate that formed was filtered off and then washed with cold acetonitrile and distilled water before being recrystallized from the appropriate solvent.

##### 4.1.1.1 6-methyl-4-phenyl-N-[4-sulfamoylphenyl]-2-thioxo-1,2,3,4-tetrahydropyrimidine -5-carboxamide (3a)

White powder (acetonitrile) (0.442 g, 55% yield), m. p: 266°C–269°C; ^1^H NMR (400 MHz, DMSO-*d*_*6*_) δ 10.06 (d, *J* = 10.2 Hz, 2H), 9.51 (s, 1H), 7.71 (s, 4H), 7.36 (t, *J* = 7.4 Hz, 2H), 7.29–7.21 (m, 5H), 5.43 (d, *J* = 3.0 Hz, 1H), 2.09 (s, 3H); ^13^C NMR DEPTQ-135 (100 MHz, DMSO-*d*_*6*_) δ 174.23 (s), 165.36 (s), 143.05 (s), 141.94 (s), 138.39 (s), 136.66 (s), 128.69 (s), 127.75 (s), 126.53 (s), 126.29 (s), 119.11 (s), 106.74 (s), 55.00 (s), 16.59 (s); MS (ESI^+^) m/z 424.6 [M + Na]^+^, 826.5 [2M + Na]^+^; MS (ESI^−^) m/z 400.7 [M-H]^-^. Anal. Calcd. For C_18_H_18_N_4_O_3_S_2_ (402.49): *C*, 53.72; H, 4.51; *N*, 13.92. Found: C, 53.61; H, 4.82; *N*, 14.04.

##### 4.1.1.2 4-[3-hydroxyphenyl]-6-methyl-N-[4-sulfamoylphenyl]-2-thioxo-1,2,3,4-tetrahydropyrimidine-5-carboxamide (3b)

White powder (acetonitrile) (0.334 g, 40% yield), m. p: 288°C–291°C; ^1^H NMR (400 MHz, DMSO-*d*_*6*_) δ 10.03 (s, 2H), 9.46 (s, 2H), 7.72 (s, 4H), 7.22 (s, 2H), 7.12 (t, *J* = 7.7 Hz, 1H), 6.65 (d, *J* = 7.9 Hz, 3H), 5.35 (d, *J* = 2.7 Hz, 1H), 2.07 (s, 3H); ^13^C NMR DEPTQ-135 (100 MHz, DMSO-*d*_*6*_) δ174.19 (s), 165.39(s), 157.60 (s), 144.59 (s), 142.00 (s), 138.35 (s), 136.39 (s), 129.60 (s), 126.53 (s), 119.13 (s), 116.74 (s), 114.70 (s), 113.15 (s), 106.88 (s), 54.97 (s), 16.56 (s); MS (ESI^+^) m/z 858.4 [2M + Na]^+^; MS (ESI^−^) m/z 416.6 [M-H]^-^, 834.5 [2M-H]^-^. Anal. Calcd. For C_18_H_18_N_4_O_4_S_2_ (418.49): C, 51.66; H, 4.34; N, 13.39. Found: C, 51.92; H, 4.50; N, 13.67.

##### 4.1.1.3 4-[3-hydroxy-4-methoxyphenyl]-6-methyl-N-[4-sulfamoylphenyl]-2-thioxo-1,2,3,4-tetrahydropyrimidine-5-carboxamide (3c)

White powder (acetic acid) (0.224 g, 25% yield), m. p: 296°C; ^1^H NMR (400 MHz, DMSO-*d*_*6*_) δ 9.99 (s, 2H), 9.42 (s, 1H), 9.02 (s, 1H), 7.72 (s, 4H), 7.22 (s, 2H), 6.86 (d, *J* = 8.4 Hz, 1H), 6.71 (d, *J* = 2.1 Hz, 1H), 6.62 (d, *J* = 8.3 Hz, 1H), 5.31 (d, *J* = 2.9 Hz, 1H), 3.72 (s, 3H), 2.08 (s, 3H); ^13^C NMR DEPTQ-135 (100 MHz, DMSO-*d*_*6*_) δ 173.85 (s), 165.40 (s), 147.33 (s), 146.59 (s), 142.03 (s), 138.31 (s), 136.28 (s), 135.86 (s), 126.51 (s), 119.09 (s), 117.01 (s), 113.79 (s), 112.11 (s), 107.00 (s), 55.67 (s), 54.65 (s), 16.54 (s); MS (ESI^+^) m/z 470.6 [M + Na]^+^, 918.3 [2M + Na]^+^; MS (ESI^−^) m/z 446.7 [M-H]^-^, 894.4 [2M-H]^-^. Anal. Calcd. For C_19_H_20_N_4_O_5_S_2_ (448.51): C, 50.88; H, 4.49; N, 12.49. Found: C, 51.18; H, 4.64; N, 12.73.

##### 4.1.1.4 6-Methyl-N-[4-sulfamoylphenyl]-2-thioxo-4-[3,4,5-trimethoxyphenyl]-1,2,3,4-tetrahydropyrimidine-5-carboxamide (3d)

White powder (ethanol) (0.384 g, 39% yield), m. p: 270°C–272°C; ^1^H NMR (400 MHz, DMSO-*d*_*6*_) δ 10.09 (s, 1H), 10.07 (s, 1H), 9.46 (s, 1H), 7.73 (s, 4H), 7.23 (s, 2H), 6.56 (s, 2H), 5.40 (d, *J* = 2.7 Hz, 1H), 3.69 (s, 6H), 3.62 (s, 3H), 2.08 (s, 3H); ^13^C NMR DEPTQ-135 (100 MHz, DMSO-*d*_*6*_) δ 174.38 (s), 165.59 (s), 153.00 (s), 141.93 (s), 138.64 (s), 138.51 (s), 137.07 (s), 136.55 (s), 126.61 (s), 119.23 (s), 106.65 (s), 103.54 (s), 60.02 (s), 55.88 (s), 54.93 (s), 16.62 (s); MS (ESI^+^) m/z 514.6 [M + Na]^+^, 1006.3 [2M + Na]^+^; MS (ESI^−^) m/z 490.6 [M-H]^-^, 982.4 [2M-H]^-^. Anal. Calcd. For C_21_H_24_N_4_O_6_S_2_ (492.57): C, 51.21; H, 4.91; N, 11.37. Found: C, 51.37; H, 5.11; N, 11.68.

##### 4.1.1.5 4-[3, 4-dimethoxyphenyl]-6-methyl-N-[4-sulfamoylphenyl]-2-thioxo-1,2,3,4-tetrahydropyrimidine-5-carboxamide (3e)

White powder (ethanol) (0.323g, 35% yield), m. p: 272°C–274°C; ^1^H NMR (400 MHz, DMSO-*d*_*6*_) δ 10.02 (s, 2H), 9.45 (s, 1H), 7.72 (s, 4H), 7.22 (s, 2H), 6.92 (d, *J* = 8.4 Hz, 1H), 6.84 (s, 1H), 6.78 (d, *J* = 10.3 Hz, 1H), 5.38 (d, *J* = 2.8 Hz, 1H), 3.71 (s, 3H), 3.67 (s, 3H), 2.09 (s, 3H); ^13^C NMR DEPTQ-135 (100 MHz, DMSO-*d*_*6*_) δ 174.05 (s), 165.48 (s), 148.68 (s), 148.43 (s), 141.97 (s), 138.39 (s), 136.54 (s), 135.40 (s), 126.54 (s), 119.12 (s), 118.31 (s), 111.88 (s), 110.37 (s), 106.74 (s), 55.57 (s), 55.43 (s), 54.60 (s), 16.58 (s); MS (ESI^+^) m/z 484.6 [M + Na]^+^, 946.2 [2M + Na]^+^; MS (ESI^−^) m/z 460.6 [M-H]^-^, 922.4 [2M-H]^-^. Anal. Calcd. For C_20_H_22_N_4_O_5_S_2_ (462.54): C, 51.94; H, 4.79; N, 12.11. Found: C, 52.24; H, 5.05; N, 12.26.

##### 4.1.1.6 6-methyl-2-oxo-4-phenyl-N-[4-sulfamoylphenyl]-1,2,3,4-tetrahydropyrimidine-5-carboxamide (3f)

White powder (methanol) (0.463 g, 60% yield), m. p: 258°C–260°C; ^1^H NMR (400 MHz, DMSO-*d*_*6*_) δ 9.88 (s, 1H), 8.82 (s, 1H), 7.71 (s, 4H), 7.66 (s, 1H), 7.37–7.19 (m, 7H), 5.44 (d, *J* = 2.4 Hz, 1H), 2.07 (s, 3H); ^13^C NMR DEPTQ-135 (100 MHz, DMSO-*d*_*6*_) δ 165.66 (s), 152.51 (s), 144.28 (s), 142.22 (s), 139.76 (s), 138.09 (s), 128.52 (s), 127.38 (s), 126.48 (s), 126.17 (s), 118.96 (s), 104.95 (s), 54.94 (s), 17.15 (s). Anal. Calcd. For C_18_H_18_N_4_O_4_S (386.43): C, 55.95; H, 4.7; N, 14.5. Found: C, 56.08; H, 4.99; N, 14.63.

##### 4.1.1.7 4-[3-hydroxyphenyl]-6-methyl-2-oxo-N-[4-sulfamoylphenyl]-1,2,3,4-tetrahydropyrimidine-5-carboxamide (3 g)

White powder (methanol) (0.539 g, 67% yield), m. p: 270°C–272°C; ^1^H NMR (400 MHz, DMSO-*d*_*6*_) δ 9.86 (s, 1H), 9.39 (s, 1H), 8.78 (s, 1H), 7.76–7.68 (m, 4H), 7.60 (s, 1H), 7.21 (s, 2H), 7.09 (t, *J* = 7.8 Hz, 1H), 6.72–6.65 (m, 2H), 6.62 (d, *J* = 8.1 Hz, 1H), 5.36 (d, *J* = 2.1 Hz, 1H), 2.05 (s, 3H); ^13^C NMR DEPTQ-135 (100 MHz, DMSO-*d*_*6*_) δ 165.69 (s), 157.52 (s), 152.61 (s), 145.88 (s), 142.30 (s), 139.54 (s), 138.06 (s), 129.44 (s), 126.49 (s), 118.99 (s), 116.64 (s), 114.31 (s), 113.02 (s), 105.12 (s), 54.85 (s), 17.14 (s). Anal. Calcd. For C_18_H_18_N_4_O_5_S (402.43): C, 53.72; H, 4.51; N, 13.92. Found: C, 53.46; H, 4.60; N, 14.20.

##### 4.1.1.8 4-[3-hydroxy-4-methoxyphenyl]-6-methyl-2-oxo-N-[4-sulfamoylphenyl]-1,2,3,4-tetrahydropyrimidine-5-carboxamide (3 h)

White powder (methanol) (0.467 g, 54% yield), m. p: 275°C–279°C; ^1^H NMR (400 MHz, DMSO-*d*_*6*_) δ 9.81 (s, 1H), 8.95 (s, 1H), 8.75 (s, 1H), 7.75–7.68 (m, 4H), 7.54 (s, 1H), 7.21 (s, 2H), 6.82 (d, *J* = 8.3 Hz, 1H), 6.73 (s, 1H), 6.62 (d, *J* = 8.3 Hz, 1H), 5.32 (d, *J* = 2.3 Hz, 1H), 3.71 (s, 3H), 2.05 (s, 3H); ^13^C NMR DEPTQ-135 (100 MHz, DMSO-*d*_*6*_) δ 166.17 (s), 152.95 (s), 147.49 (s), 146.98 (s), 142.79 (s), 139.83 (s), 138.48 (s), 137.62 (s), 126.93 (s), 119.42 (s), 117.21 (s), 114.12 (s), 112.52 (s), 105.74 (s), 56.13 (s), 55.00 (s), 17.58 (s). Anal. Calcd. For C_19_H_20_N_4_O_6_S (432.45), C, 52.77; H, 4.66; N, 12.96. Found, C, 52.99; H, 4.75; N, 13.18.

##### 4.1.1.9 6-methyl-2-oxo-N-[4-sulfamoylphenyl]-4-[3,4,5-trimethoxyphenyl]-1,2,3,4-tetrahydropyrimidine-5-carboxamide (3i)

White powder (methanol) (0.476 g, 50% yield), m. p: 260°C–263°C; ^1^H NMR (400 MHz, DMSO-*d*_*6*_) δ 9.90 (s, 1H), 8.78 (s, 1H), 7.72 (s, 4H), 7.60 (s, 1H), 7.21 (s, 2H), 6.56 (s, 2H), 5.39 (d, *J* = 2.3 Hz, 1H), 3.68 (s, 6H), 3.60 (s, 3H), 2.05 (s, 3H); ^13^C NMR DEPTQ-135 (100 MHz, DMSO-*d*_*6*_) δ 165.86 (s), 152.87 (s), 152.48 (s), 142.19 (s), 139.78 (s), 139.54 (s), 138.19 (s), 136.80 (s), 126.52 (s), 119.02 (s), 104.73 (s), 103.40 (s), 59.97 (s), 55.83 (s), 54.95 (s), 17.14 (s); MS (ESI^+^) m/z 498.6 [M + Na]^+^, 974.3 [2M + Na]^+^; MS (ESI^−^) m/z 474.6 [M-H]^-^. Anal. Calcd. For C_21_H_24_N_4_O_7_S (476.5): C, 52.93; H, 5.08; N, 11.76. Found: C, 53.03; H, 5.33; N, 11.87.

##### 4.1.1.10 4-[3, 4-dimethoxyphenyl]-6-methyl-2-oxo-N-[4-sulfamoylphenyl]-1,2,3,4-tetrahydropyrimidine-5-carboxamide (3j)

White powder (ethanol) (0.420 g, 47% yield), m. p: 266°C–267°C; ^1^H NMR (400 MHz, DMSO-*d*_*6*_) δ 9.84 (s, 1H), 8.77 (s, 1H), 7.71 (s, 4H), 7.58 (s, 1H), 7.21 (s, 2H), 6.90 (d, *J* = 8.3 Hz, 1H), 6.86 (s, 1H), 6.80 (d, *J* = 8.3 Hz, 1H), 5.39 (d, *J* = 2.4 Hz, 1H), 3.71 (s, 3H), 3.66 (s, 3H), 2.06 (s, 3H); ^13^C NMR DEPTQ-135 (100 MHz, DMSO-*d*_*6*_) δ 165.78 (s), 152.48 (s), 148.63 (s), 148.17 (s), 142.25 (s), 139.60 (s), 138.10 (s), 136.67 (s), 126.49 (s), 118.96 (s), 118.09 (s), 111.78 (s), 110.31 (s), 104.96 (s), 55.55 (s), 55.41 (s), 54.56 (s), 17.14 (s); MS (ESI^+^) m/z 468.7 [M + Na]^+^, 914.5 [2M + Na]^+^; MS (ESI^−^) m/z 444.8 [M-H]^-^. Anal. Calcd. For C_20_H_22_N_4_O_6_S (446.48), C, 53.8; H, 4.97; N, 12.55. Found, C, 53.63; H, 5.13; N, 12.68.

### 4.2 Biology

#### 4.2.1 Microsomal PGES-1 (mPGES-1) enzyme assay

Microsomal measures of A549 cells expressing mPGES-1 were made in accordance with prior research findings (Koeberle et al., 2008). These cells were cultivated in Dulbecco’s Modified Eagle Medium and resuspended in a homogenization buffer. Subsequently, the microsomes were subjected to pre-incubation with either assessed compounds or a carrier solution containing 0.1 percent DMSO. The enzymatic process was halted by introducing FeCl_3_, citric acid, and 11-PGE_2_ as an internal standard. The quantities of Prostaglandin E_2_ (PGE_2_) were measured via RP-HPLC methodologies.

#### 4.2.2 5-LOX enzyme assay

The study used an enzyme immune assay (EIA) kit (catalogue no. 760700, Cayman Chemical, Ann Arbor, Michigan, USA) to evaluate the inhibitory activity of target analogues against soya bean 5-LOX, ensuring compliance with manufacturer’s instructions and protocols, and calculating IC_50_ values (Roschek et al., 2009).

#### 4.2.3 *In vivo* anti-inflammatory assay

Compounds **3c**, **3e**, **3h**, and **3j** were chosen for *in vivo* anti-inflammatory testing using the carrageen-induced paw edema bioassay method described by Winter et al, (1962). The compounds’ efficacy was measured as edema inhibition percentage (EI%) after 1, 3, and 5 h of carrageenan injection vs. the conventional medicine Celecoxib.

#### 4.2.4 Effect on inflammatory cytokines

In this study, specializing ELISA kits were used to determine the concentration of inflammatory cytokines PGE_2_, IL-6, and TNF-α. The study’s findings were examined in accordance with the instructions provided by the manufacturer, and measurements were taken based on the optical density at 450 nm.

#### 4.2.5 Ulcerogenic effect assay

The ulcerogenic effects of compounds **3e** and **3j** were evaluated by macroscopic examination of rat intestinal mucosa after oral administration of 10 mg/kg of these compounds and indomethacin and celecoxib (Manivannan and Chaturvedi, 2011). See Appendix A for details.

### 4.3 Molecular docking

#### 4.3.1 Ligand structure generation

OpenBabel v.3.1.1 (O’Boyle et al., 2011) was used to convert the structures’ SMILES codes to three-dimensional configurations that were subsequently subjected to a minimization of energy using the steepest descent technique with the same software. The minimization was performed by the force field MMFF94. Using AutoDockTools v.4.2, all torsions of the selected structures were assigned and their Gasteiger charges were provided for all studied atoms in structures (Morris et al., 2009).

#### 4.3.2 Protein structure preparation

For docking screening, the mPGES-1 and 5-LO crystal structures (PDB codes: 4bpm and 6n2w, respectively) (Li et al., 2014; Koeberle and Werz, 2018) were used. PDBfixer (Eastman et al., 2013) was used to edit the downloaded structure, adding missing residues and atoms, and removing co-crystalized H_2_O and heteroatoms. Through AutoDock Tools v.4.2, polar hydrogen and Gasteiger charges were subsequently made available for both proteins.

#### 4.3.3 Structural docking

The docking process was carried out using the PyRx platform’s built-in AutoDock Vina software (Eastman et al., 2013; Dallakyan et al., 2015). According to the co-crystalized ligands of both enzymes, the docking search grid boxes were determined to perfectly enclose them with a 20 Å^3^ total size.

The grid box’s coordinates were set to be *x* = −9.682; *y* = 4.274; *z* = −23.145 and *x* = 45.424; *y* = 92.375; *z* = 34.811, respectively. The level of exhaustion was held at 24. Ten poses were generated for each docking experiment. Docking poses were analyzed and visualized using Pymol software (Seeliger and de Groot, 2010).

### 4.4 Molecular dynamics simulations

The NAMD 3.0.0 program, which makes use of the Charmm-36 force field, was used to do molecular dynamics simulations (Phillips et al., 2005; Ribeiro et al., 2018). The QwikMD toolbox in VMD software was used to build protein systems (Humphrey et al., 1996). The procedure encompassed the examination of the protein structure to identify any hydrogens that were absent, the modification of the protonation states of the amino acids to achieve a pH of 7.4, and the elimination of co-crystallized water molecules. Following this, the entire configuration was enclosed within an orthorhombic container including TIP3P water molecules, along with the addition of sodium (Na^+^) and chloride (Cl-) ions at a concentration of 0.15 M, creating a solvent buffer with a size of 20 Å. Subsequently, the constructed systems underwent energy minimization and equilibration for a duration of 5 nanoseconds. In the context of protein-ligand complexes, the initial configurations with the highest scores were utilized as a basis for subsequent simulation. The VMD plugin Force Field Toolkit (ffTK) was utilized to calculate the properties and topologies of the compounds. Subsequently, the resulting parameters and topology files were introduced into VMD to facilitate the accurate reading of the protein-ligand complexes and subsequent execution of the simulation procedures.

### 4.5 Binding free energy calculations

The Molecular Mechanics Poisson-Boltzmann Surface Area (MM-PBSA) technique, which was introduced into the AMBER18 MMPBSA. py module, was used to estimate the binding free energy for the docked complex. The results of this calculation may be found in the following sentence (Miller et al., 2012). The trajectories were processed into one hundred frames, and the net energy of the system was predictable using the below formula:

$$\Delta G_{\text{Binding}}=\Delta G_{\text{Complex}}\;–\;\Delta G_{\text{Receptor}}\;–\;\Delta G_{\text{Inhibitor}}$$

In order to accurately compute each of the previously mentioned variables, it is necessary to consider a wide variety of energy components. Some of these components include electrostatic energy, van der Waals energy, the polar contribution to solvation energy, as well as the internal energy derived from molecular mechanics.

## Data Availability

The original contributions presented in the study are included in the article/supplementary material, further inquiries can be directed to the corresponding authors.
